# Supplementary material for: Reconfiguration of the reductive TCA cycle enables high-level succinic acid production by Yarrowia lipolytica
Source: Nat Commun. 2023 Dec 20;14:8480. doi: 10.1038/s41467-023-44245-4 (PMC10733433; doi:10.1038/s41467-023-44245-4)
Supplement: Supplementary file 1 — Supplementary Information [file 41467_2023_44245_MOESM1_ESM.pdf]

Reconfiguration of the reductive TCA cycle enables high-level succinic acid production by *Yarrowia lipolytica*

Cui *et al.*

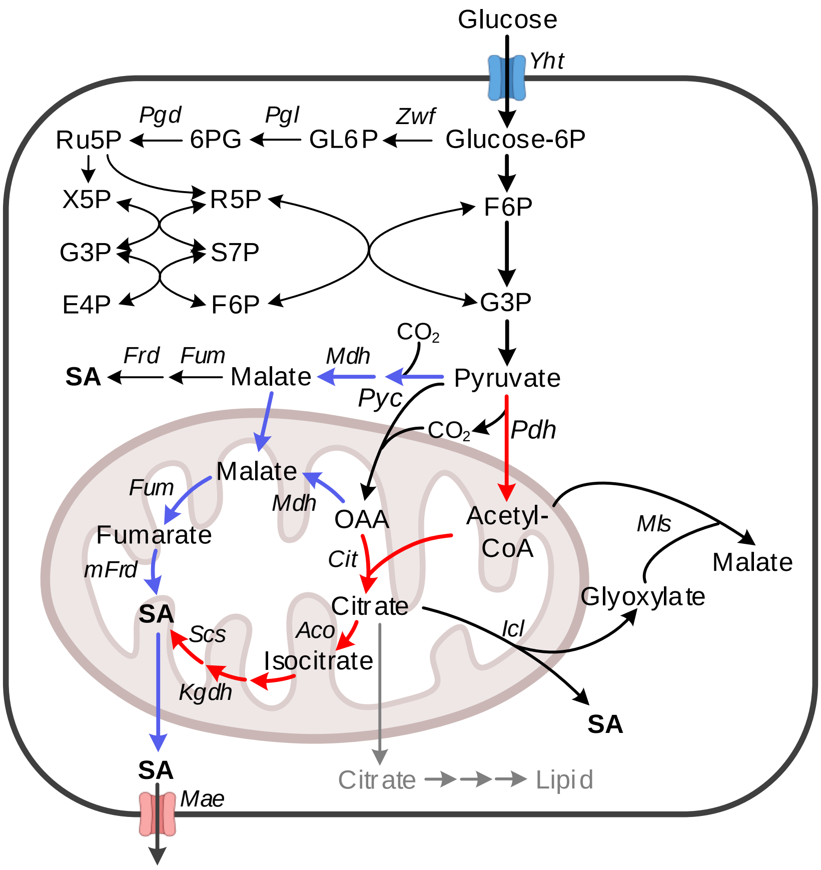

Supplementary Fig. 1. Schematic illustration of all SA biosynthesis related metabolic pathway in *Yarrowia lipolytica*. Red arrows represent the oxidative TCA cycle, and blue arrows represent the reductive TCA cycle. Glucose transport: hexose transporter (encoding by *Yht*); Pentose phosphate pathway: 6-phosphate-glucose dehydrogenase (encoding by *Zwf*); 6-phospho-gluconolactonase (encoding by *Pgl*); 6-phospho-gluconate dehydrogenase (encoding by *Pgd*); Oxidative TCA pathway: pyruvate dehydrogenase (encoding by *Pdh*); citrate synthetase (encoding by *Cit*); aconitate hydratase (encoding by *Aco*); α-ketoglutarate dehydrogenase (encoding by *Kgdh*); succinyl-CoA synthetase (encoding by *Scs*); Reductive TCA pathway: pyruvate carboxylase (*Pyc*); malate dehydrogenase (encoding by *Mdh*); fumarate hydratase (encoding by *Fum*); fumarate reductase (encoding by *Frd*); mitochondrial fumarate reductase (*mFrd*); Glyoxylate bypass: isocitrate lyase (encoding by *Icl*); malate synthetase (encoding by *Mls*); SA transport: dicarboxylic acid transporter (encoding by *Mae*).

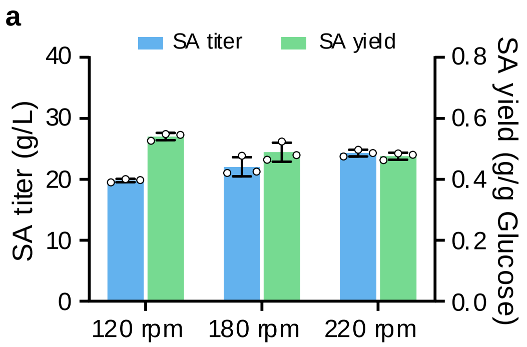

Supplementary Fig. 2. Effect of shaking speeds on SA production of different Y. lipolytica strains. Comparison of SA production performance of PGC91 (**a**) and PGC91-TbFrd (**b**) strains in YPD media with different shaking speeds. Data are presented as mean ± s.e.m. (n = 3 biologically independent samples). The initial concentration of glucose was 60 g/L. Source data are provided as a Source Data file.

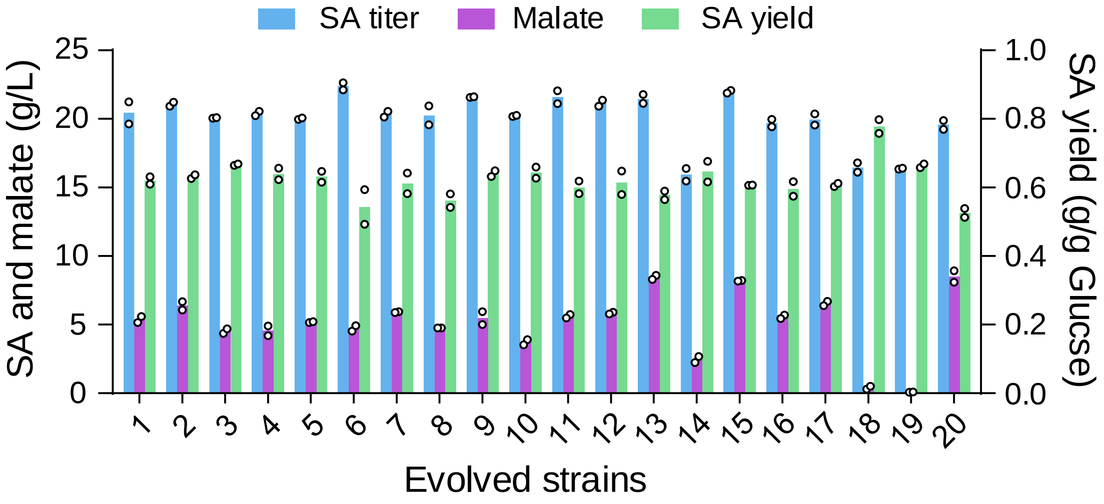

Supplementary Fig. 3. Comparison of SA production and by-product accumulation in different evolved strains. Data are presented as mean ± s.e.m. (n = 2 biologically independent samples). The initial concentration of glucose was 60 g/L. Source data are provided as a Source Data file.

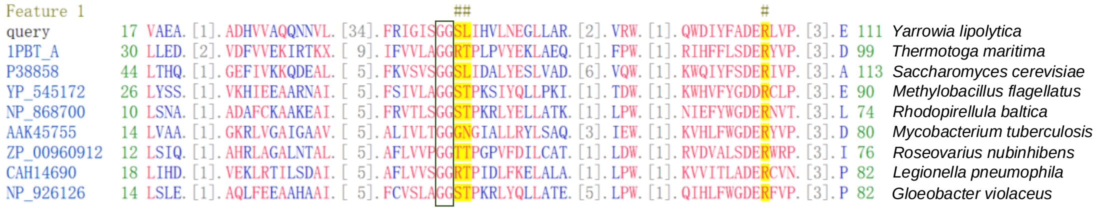

Supplementary Fig. 4. Amino acid sequence alignment of Pgl from different species. The amino acid residues of active center are marked by "#" and yellow highlight. Box represents the conservative glycine residues.

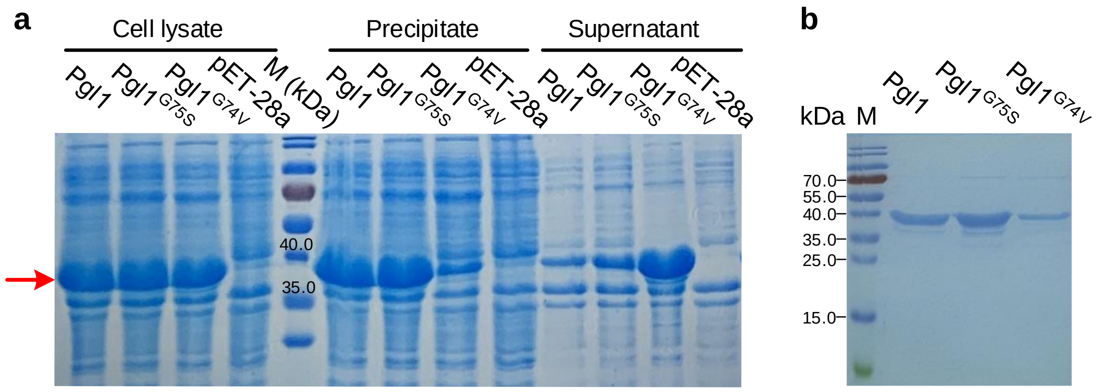

Supplementary Fig. 5. Heterologous expression and purification of YlPgl1 and its mutants in *E. coli*. a Analysis of protein expression via SDS-PAGE. **b** Analysis of purified target proteins via SDS-PAGE. M, Protein molecular weight marker. Red arrow indicates the bands of target protein. Source data are provided as a Source Data file.

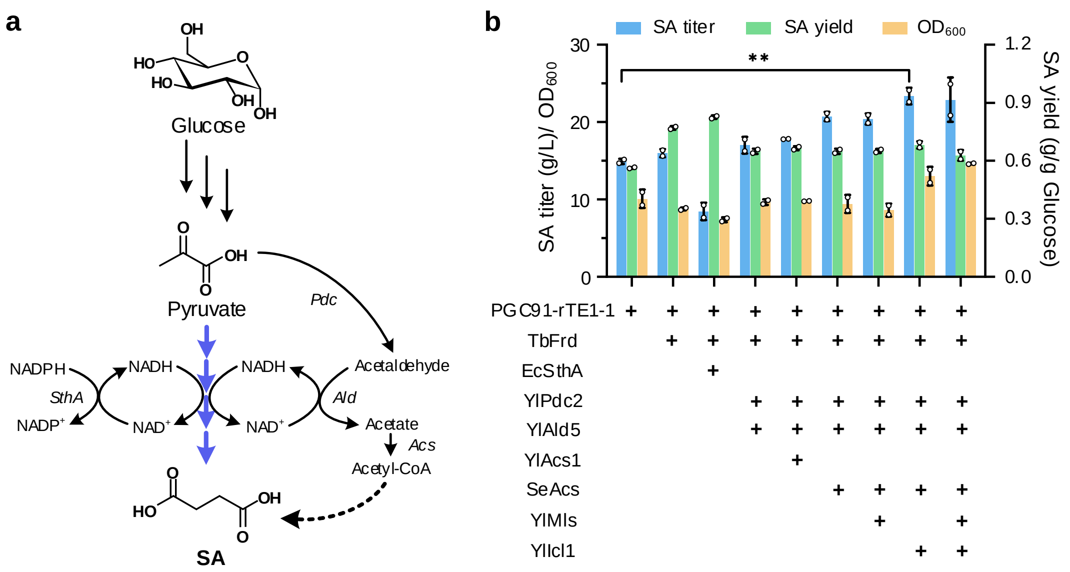

Supplementary Fig. 6. Increasing cytoplasmic NADH supply for SA synthesis. a Schematic diagram of metabolic engineering strategies to improve cytoplasmic NADH regeneration in *Y. lipolytica*. Blue arrows represent the reductive TCA cycle. **b** Effects of transhydrogenase and pyruvate decarboxylase bypass overexpression on SA production of PGC91-rTE1-1 strain. The initial concentration of glucose was 60 g/L. Data are presented as mean ± s.e.m. (n = 2 biologically independent samples). *EcSthA* encoding *E. coil* transhydrogenase, *YlPdc2* encoding pyruvate decarboxylase, *Ylald5* encoding aldehyde dehydrogenase, *YlAcs1* encoding endogenous acetyl-CoA synthetase, *SeAcs* encoding *Salmonella enteric* acetyl-CoA synthetase, *YlMls* encoding malate synthetase, *YlIcl1* encoding isocitrate lyase. Statistical analysis was carried out by using Student's *t*-test (one-tailed; two-sample unequal variance; *P < 0.05, **P < 0.01, ***P < 0.001). Source data are provided as a Source Data file.

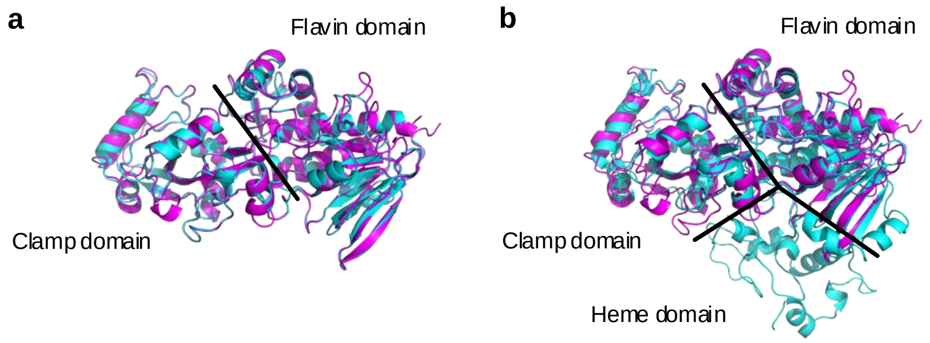

Supplementary Fig. 7. The protein structure similarity between the NAD(P) (+)-binding domains of different fumarate reductases. **a** ScOsm1 derived from *Saccharomyces cerevisiae*. **b** SfFcc3 derived from *Shewanella frigidimarina*. Purple structure stand for TbFrd from *Trypanosoma brucei*. Source data are provided as a Source Data file.

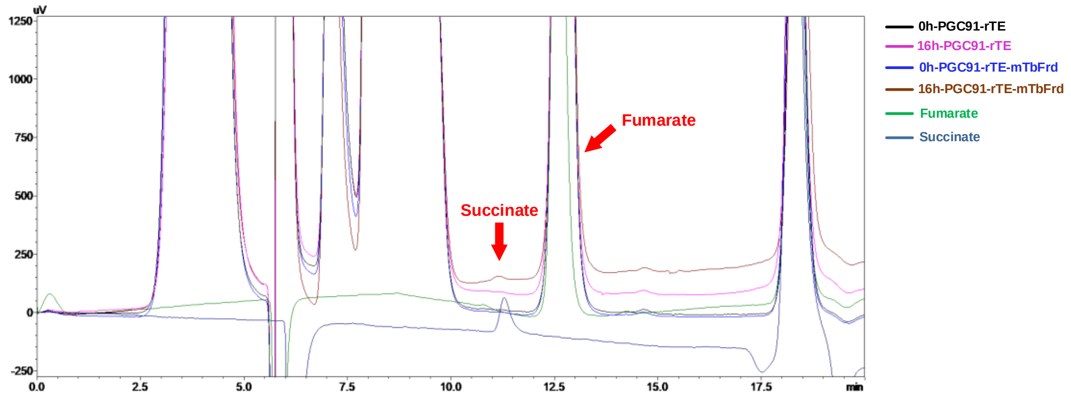

Supplementary Fig. 8. Fumarate reductase activity in purified mitochondria from PGC91-rTE1-1 and PGC91-rTE-mTbFrd cells. The red arrows indicate the retention times of succinate and fumarate. The fumarate reduction reaction was initiated with 10 mM fumarate and 1 mM NADH, and monitored through the production of succinate over time. 10 mg/L succinate and 1 g/L fumarate were used as standards for HPLC detection.

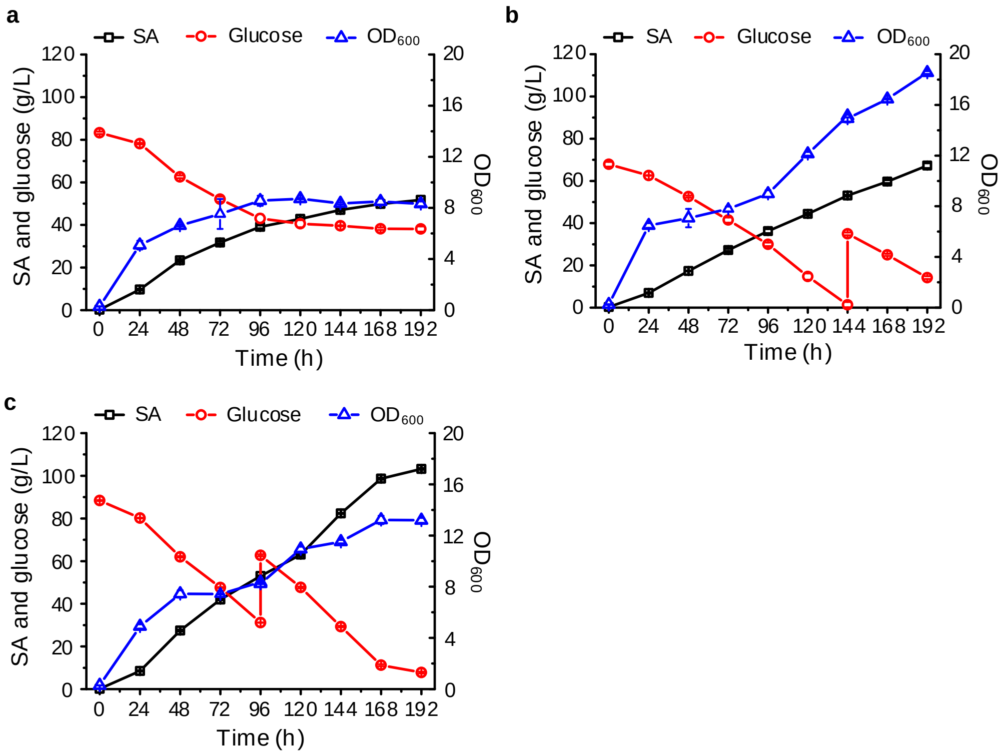

Supplementary Fig. 9. Shaking-flasks fermentation comparison of different SA high-producing strains. Long-term shaking flasks fermentation of strains Hi-SA0 (**a**), Hi-SA1 (**b**), and Hi-SA2 (**c**). The cultivation conditions were set as 30°C, 120 rpm, the initial concentration of glucose was about 80 g/L. culture samples were taken every 12 h to detect glucose, OD_{600}, and SA production. Data are presented as mean ± s.e.m. (n = 3 biologically independent samples). Source data are provided as a Source Data file.

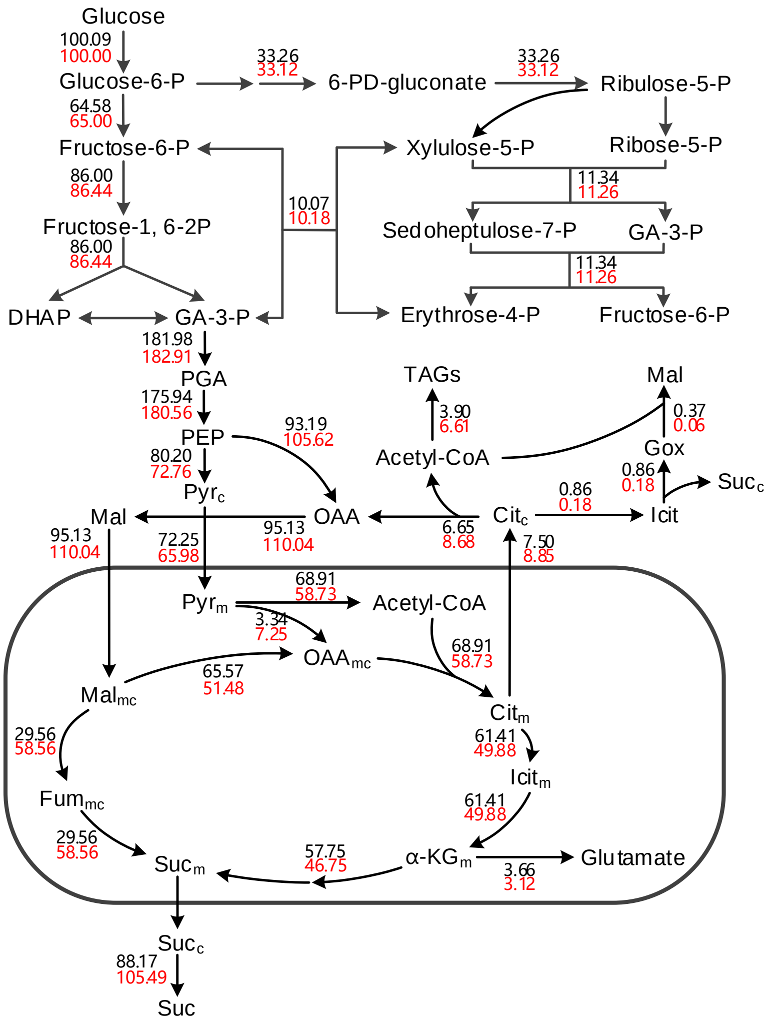

Supplementary Fig. 10. Flux distributions from ^{13}C-metabolic flux analysis. Two flux values are listed for the engineered strain Hi-SA2 cultivated at 220 rpm (top, black font) and 120 rpm (bottom, red font). Source data are provided as a Source Data file.

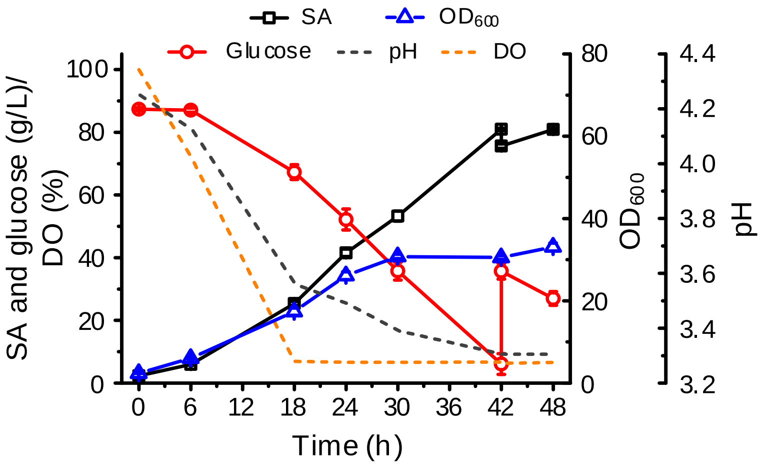

Supplementary Fig. 11. Fed-batch fermentation of Hi-SA2 strain in 5-L bioreactor with the media of YPD. DO, dissolved oxygen. Data are presented as mean ± s.e.m. (n = 2 biologically independent samples). Source data are provided as a Source Data file.

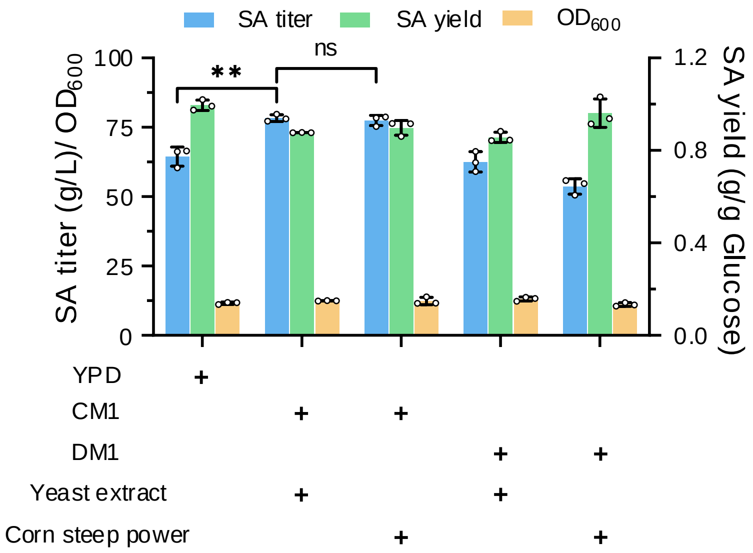

Supplementary Fig. 12. Optimizing fermentation media for SA production of Hi-SA2 strain in shaking-flasks at 120 rpm. Data are presented as mean ± s.e.m. (n = 3 biologically independent samples). The initial concentration of glucose was 80 g/L. Statistical analysis was carried out by using Student's *t*-test (one-tailed; two-sample unequal variance; *P < 0.05, **P < 0.01, ***P < 0.001; ns represents no significant difference). Source data are provided as a Source Data file.

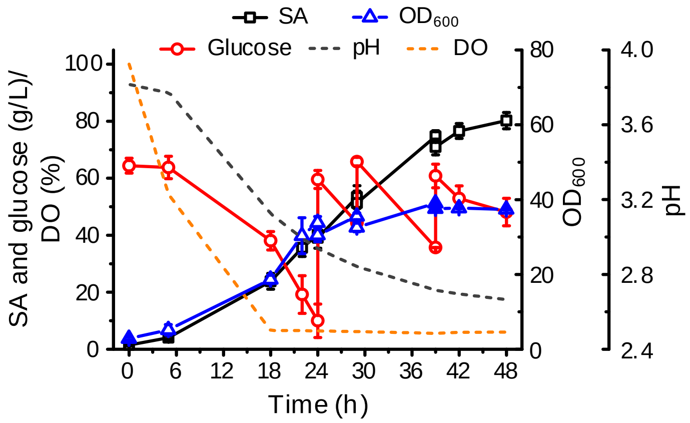

Supplementary Fig. 13. Fed-batch fermentation of Hi-SA2 strain in 5-L bioreactor with the media of modified CM1. DO, dissolved oxygen. Data are presented as mean ± s.e.m. (n = 2 biologically independent samples). Source data are provided as a Source Data file.

Supplementary Table 1. Bio-SA production by different microbial chassis.

Strain	Carbon source	Fermentation mode	Titer (g/L)	Yield (g/g)	Productivity (g/L/h)	Reference
M. succiniciproducens PALKmsmdh^{G11Q}	Glucose and glycerol	High-inoculum fed-batch, pH was maintained at 6.5	134.25	0.82	10.3	[1]
E. coli FMME-N-30	Glucose	Fed-batch, pH was maintained at 6.5	119.0	1.1	1.66	[2]
S. cerevisiae SUC-297	Glucose	Fed-batch, pH was maintained at 3.0	43.0	–	0.45	[3]
P. kudriavzevii 13723	Glucose	Batch, pH was maintained at 3.0	48.2	0.45	0.97	[4]
I. orientalis 257	Glucose	Batch, pH was buffered by $CaCO_3$	89.0	0.80	0.93	[5]
I. orientalis g3473Δ/PaGDH-DAK/g3837Δ	Glucose and glycerol	Fed-batch, pH was maintained at 3.0	109.5	0.65	0.54	[6]
Y. lipolytica PGC202	Glycerol	Fed-batch, without pH control	110.7	0.52	0.80	[7]
Y. lipolytica ST8578	Glucose	Fed-batch, pH was maintained at 5.0	35.3	0.26	0.61	[8]
Y. lipolytica PGC62-SYF-Mae	Glucose	Fed-batch, pH was maintained at 5.5	101.4	0.37	0.70	[9]
Y. lipolytica Hi-SA2	Glucose	Fed-batch in 5-L YPD medium, without pH adjustment	80.9	0.90	1.69	This study
Y. lipolytica Hi-SA2	Glucose	Fed-batch in 50-L CM1 medium, without pH adjustment	111.9	0.79	1.79	This study

Supplementary Table 2. Screen of the soluble fumarate reductases from different species for SA production in *Y. lipolytica*.

Name	Resource	Reference sequence	Encoding products	Amino acids number
TbFrd	*Trypanosoma brucei*	AAN40014.1	NADH-dependent fumarate reductase	1142
ScOsm1	*Saccharomyces cerevisiae*	NP_012585	$FADH_2$-dependent fumarate reductase	501
LmFrd	*Leishmania major*	XP_003722478.1	NADH-dependent fumarate reductase-like protein	1194
TcFrd	*Trypanosomacruzi*	XP_807320.1	NADH-dependent fumarate reductase	1215
LdFrd	*Leishmania donovani*	XP_003864704.1	Putative NADH-dependent fumarate reductase	1147
SfFcc3	*Shewanella frigidimarina*	WP_041413240.1	Cytochrom_C3/ Flavo_cyto_c domain-containing protein	596

Supplementary Table 3. Genes used in this study and their functions and sources.

Name	Encoding product	Sources	Reference sequence	Reference
TbFrd	NADH-dependent fumarate reductase	*T. brucei*	AAN40014.1	[10]
EcFum	Fumarate hydratase	*E. coil*	b1611	This study
YlFum	Fumarate hydratase	*Y. lipolytica*	YALI0C06776p	This study
YlMdh1	Malate dehydrogenase	*Y. lipolytica*	YALI0D16753g	This study
YlMdh2	Malate dehydrogenase	*Y. lipolytica*	YALI0E14190g	This study
YlMls	Malate synthetase	*Y. lipolytica*	YALI0D19140p	This study
YlIcl1	Isocitrate lyase	*Y. lipolytica*	YALI0C16885g	This study
YlYht1	Hexose transporter	*Y. lipolytica*	YALI0C06424p	[11]
YlYht4	Hexose transporter	*Y. lipolytica*	YALI0E23287p	[11]
EcSthA	Transhydrogenase	*E. coil*	b3962	[5]
YlPdc2	Pyruvate decarboxylase	*Y. lipolytica*	YALI0D10131g	[12]
YlAld5	Aldehyde dehydrogenase	*Y. lipolytica*	YALI0E00264g	[12]
YlAcs1	Acetyl-CoA synthetase	*Y. lipolytica*	YALI0F05962g	[12]
$SeAcs^{L641P}$	Acetyl-CoA synthetase	*Salmonella enteric*	WP_000083882.1	[13]
YlPgl1	6-phospho-gluconolactonase	*Y. lipolytica*	YALI0C19085g	This study
CgMdh	Malate dehydrogenase	*Corynebacterium glutamicum*	Cgl2380	[1]
SpMae1	Dicarboxylic acid transporter	*Schizosaccharomyces pombe*	SPAPB8E5.03	[9]
YlKgdh	α-Ketoglutarate dehydrogenase	*Y. lipolytica*	YALI0E33517p	[7]
YlScs2	Succinyl-CoA synthetase	*Y. lipolytica*	YALI0D04741g	[7]

Supplementary references

1. Ahn, J.H. *et al.* Enhanced succinic acid production by *Mannheimia* employing optimal malate dehydrogenase. *Nat Commun* **11** (2020).
2. Gao, C. *et al.* Improving succinate production by engineering oxygen-dependent dynamic pathway regulation in *Escherichia coli*. *Systems Microbiology and Biomanufacturing* **2**, 331-344 (2022).
3. Ahn, J.H., Jang, Y.S. & Lee, S.Y. Production of succinic acid by metabolically engineered microorganisms. *Curr Opin Biotech* **42**, 54-66 (2016).
4. Rush, B.J. & Fosmer, A.M. Methods for succinate production. (Bioamber Incorporated, 2018).
5. Rush, B.J. *et al.* Yeast cells having reductive TCA pathway from pyruvate to succinate and overexpressing an exogenous NAD(P) plus transhydrogenase enzyme. (Cargill Incorporated, 2021).
6. Tran, V.G. *et al.* An end-to-end pipeline for succinic acid production at an industrially relevant scale using *Issatchenkia orientalis*. *bioRxiv*, 2023.2004.2030.538856 (2023).
7. Cui, Z.Y. *et al.* Engineering of unconventional yeast *Yarrowia lipolytica* for efficient succinic acid production from glycerol at low pH. *Metab Eng* **42**, 126-133 (2017).
8. Babaei, M. *et al.* Engineering oleaginous yeast as the host for fermentative succinic acid production from glucose. *Front Bioeng Biotech* **7** (2019).
9. Jiang, Z.N. *et al.* Engineering of *Yarrowia lipolytica* transporters for high-efficient production of biobased succinic acid from glucose. *Biotechnol Biofuels* **14** (2021).
10. Yang, L., Lubeck, M., Ahring, B.K. & Lubeck, P.S. Enhanced succinic acid production in *Aspergillus saccharolyticus* by heterologous expression of fumarate reductase from Trypanosoma brucei. *Appl Microbiol Biot* **100**, 1799-1809 (2016).
11. Hapeta, P. *et al.* The role of hexokinase and hexose transporters in preferential

use of glucose over fructose and downstream metabolic pathways in the yeast *Yarrowia lipolytica*. *Int J Mol Sci* **22** (2021).

12. Markham, K.A. *et al.* Rewiring *Yarrowia lipolytica* toward triacetic acid lactone for materials generation. *P Natl Acad Sci USA* **115**, 2096-2101 (2018).
13. Gao, C.J. *et al.* Robust succinic acid production from crude glycerol using engineered *Yarrowia lipolytica*. *Biotechnol Biofuels* **9** (2016).
