# Supplementary material for: Reconfiguration of the reductive TCA cycle enables high-level succinic acid production by Yarrowia lipolytica
Source: Nat Commun. 2023 Dec 20;14:8480. doi: 10.1038/s41467-023-44245-4 (PMC10733433; doi:10.1038/s41467-023-44245-4)
Supplement: Supplementary file 3 — Description of Additional Supplementary Files [file 41467_2023_44245_MOESM3_ESM.pdf]

Description of Additional Supplementary Files

File Name: Supplementary Data 1
Description: Mutations identified in the evolution, related to Fig. 2f

File Name: Supplementary Data 2
Description: Strains and plasmids used in this study

File Name: Supplementary Data 3
Description: List of primers used in this study

File Name: Supplementary Data 4
Description: Sequences of the codon optimized genes used in this study
