# Supplementary material for: Reconfiguration of the reductive TCA cycle enables high-level succinic acid production by Yarrowia lipolytica
Source: Nat Commun. 2023 Dec 20;14:8480. doi: 10.1038/s41467-023-44245-4 (PMC10733433; doi:10.1038/s41467-023-44245-4)
Supplement: Supplementary file 8 — Reporting Summary [file 41467_2023_44245_MOESM8_ESM.pdf]

nature portfolio

Corresponding author(s): Qingsheng Qi, Jin Hou

Last updated by author(s): Dec 1, 2023

Reporting Summary

Nature Portfolio wishes to improve the reproducibility of the work that we publish. This form provides structure for consistency and transparency in reporting. For further information on Nature Portfolio policies, see our Editorial Policies and the Editorial Policy Checklist.

Statistics

For all statistical analyses, confirm that the following items are present in the figure legend, table legend, main text, or Methods section.

n/a	Confirmed	
☐	☒	The exact sample size (*n*) for each experimental group/condition, given as a discrete number and unit of measurement
☐	☒	A statement on whether measurements were taken from distinct samples or whether the same sample was measured repeatedly
☐	☒	The statistical test(s) used AND whether they are one- or two-sided<br>*Only common tests should be described solely by name; describe more complex techniques in the Methods section.*
☒	☐	A description of all covariates tested
☒	☐	A description of any assumptions or corrections, such as tests of normality and adjustment for multiple comparisons
☐	☒	A full description of the statistical parameters including central tendency (e.g. means) or other basic estimates (e.g. regression coefficient) AND variation (e.g. standard deviation) or associated estimates of uncertainty (e.g. confidence intervals)
☐	☒	For null hypothesis testing, the test statistic (e.g. *F*, *t*, *r*) with confidence intervals, effect sizes, degrees of freedom and *P* value noted<br>*Give P values as exact values whenever suitable.*
☒	☐	For Bayesian analysis, information on the choice of priors and Markov chain Monte Carlo settings
☒	☐	For hierarchical and complex designs, identification of the appropriate level for tests and full reporting of outcomes
☒	☐	Estimates of effect sizes (e.g. Cohen's *d*, Pearson's *r*), indicating how they were calculated

Our web collection on statistics for biologists contains articles on many of the points above.

Software and code

Policy information about availability of computer code

Data collection	Gas Chromatography-Mass Spectrometer (GC-MS); High pressure liquid chromatography (HPLC, Shimadzu); Spectrophotometer (Shimadzu); Qubit 2.0 Fluorometer, Eclipse 80i microscope.
Data analysis	The following commercial software was used for data analysis: OriginPro 8.5, GraphPad Prism 8.0.2, Microsoft Excel 2018, LabSolutions LC Workstation Ver.5, PyMOL 2.1, 13CFLUX2.

For manuscripts utilizing custom algorithms or software that are central to the research but not yet described in published literature, software must be made available to editors and reviewers. We strongly encourage code deposition in a community repository (e.g. GitHub). See the Nature Portfolio guidelines for submitting code & software for further information.

Data

Policy information about availability of data

All manuscripts must include a data availability statement. This statement should provide the following information, where applicable:

- Accession codes, unique identifiers, or web links for publicly available datasets
- A description of any restrictions on data availability
- For clinical datasets or third party data, please ensure that the statement adheres to our policy

The main data supporting the findings of this study are available within the article and its Supplementary Information files. Genome sequence data of evolved Y.

lipolytica strains were accessed under the BioProject accession PRJNA998929 [https://www.ncbi.nlm.nih.gov/bioproject/PRJNA998929]. Source data are provided with this paper.

Research involving human participants, their data, or biological material

Policy information about studies with human participants or human data. See also policy information about sex, gender (identity/presentation), and sexual orientation and race, ethnicity and racism.

Reporting on sex and gender	Not applicable
Reporting on race, ethnicity, or other socially relevant groupings	Not applicable
Population characteristics	Not applicable
Recruitment	Not applicable
Ethics oversight	Not applicable

Note that full information on the approval of the study protocol must also be provided in the manuscript.

Field-specific reporting

Please select the one below that is the best fit for your research. If you are not sure, read the appropriate sections before making your selection.

☒ Life sciences ☐ Behavioural & social sciences ☐ Ecological, evolutionary & environmental sciences

For a reference copy of the document with all sections, see nature.com/documents/nr-reporting-summary-flat.pdf

Life sciences study design

All studies must disclose on these points even when the disclosure is negative.

Sample size	Generally, three biological replicates were adopted for data analysis due to relatively small deviation of microbial cells, which is also standard in our field that is deeded sufficient. For some special experiments, like the preliminary SA fermentation of engineering strains, fewer sample sizes are adopted, as stated in the figure legends.
Data exclusions	No data were excluded from the analysis.
Replication	Succinic acid fermentations using shake flasks were performed with biological triplicates or duplicates. Fermentations in bioreactors was performed with a three replicates. Enzyme activity assay were performed with biological triplicates. Succinic acid recovery, crystallization, and purification were performed with biological duplicates. Metabolic flux analysis were performed with biological triplicates. All attempts at replication were successful.
Randomization	After transformation, ten yeast colonies were randomly picked to select engineered strains with higher SA titer and yield by shaking flasks fermentation. Then the fermentation of high-SA producing strains was repeated. All transformation colonies were allocated into experimental groups, and the started strain was allocated into control group.
Blinding	Not relevant because no group allocation was involved in this study.

Reporting for specific materials, systems and methods

We require information from authors about some types of materials, experimental systems and methods used in many studies. Here, indicate whether each material, system or method listed is relevant to your study. If you are not sure if a list item applies to your research, read the appropriate section before selecting a response.

Materials & experimental systems

n/a	Involved in the study
☒	☐ Antibodies
☒	☐ Eukaryotic cell lines
☒	☐ Palaeontology and archaeology
☒	☐ Animals and other organisms
☒	☐ Clinical data
☒	☐ Dual use research of concern
☒	☐ Plants

Methods

n/a	Involved in the study
☒	☐ ChIP-seq
☒	☐ Flow cytometry
☒	☐ MRI-based neuroimaging
